# An Integrated Lightweight Neural Network Design and FPGA-Accelerated Edge Computing for Chili Pepper Variety and Origin Identification via an E-Nose

**DOI:** 10.3390/foods14152612

**Published:** 2025-07-25

**Authors:** Ziyu Guo, Yong Yin, Haolin Gu, Guihua Peng, Xueya Wang, Ju Chen, Jia Yan

**Affiliations:** 1 College of Artificial Intelligence, Southwest University, Chongqing 400715, China; gzy_1203@163.com (Z.G.); guhaolin@email.swu.edu.cn (H.G.); 2 Chili Pepper Research Institute, Guizhou Academy of Agricultural Sciences, Guiyang 550006, China; yinyong24@126.com (Y.Y.); 18286039217@139.com (G.P.); wangxueya1231@163.com (X.W.); juchen0924@163.com (J.C.)

**Keywords:** e-nose, FPGA-accelerated, lightweight CNN, chili pepper, variety identification, origin tracing

## Abstract

A chili pepper variety and origin detection system that integrates a field-programmable gate array (FPGA) with an electronic nose (e-nose) is proposed in this paper to address the issues of variety confusion and origin ambiguity in the chili pepper market. The system uses the AIRSENSE PEN3 e-nose from Germany to collect gas data from thirteen different varieties of chili peppers and two specific varieties of chili peppers originating from seven different regions. Model training is conducted via the proposed lightweight convolutional neural network ChiliPCNN. By combining the strengths of a convolutional neural network (CNN) and a multilayer perceptron (MLP), the ChiliPCNN model achieves an efficient and accurate classification process, requiring only 268 parameters for chili pepper variety identification and 244 parameters for origin tracing, with 364 floating-point operations (FLOPs) and 340 FLOPs, respectively. The experimental results demonstrate that, compared with other advanced deep learning methods, the ChiliPCNN has superior classification performance and good stability. Specifically, ChiliPCNN achieves accuracy rates of 94.62% in chili pepper variety identification and 93.41% in origin tracing tasks involving Jiaoyang No. 6, with accuracy rates reaching as high as 99.07% for Xianjiao No. 301. These results fully validate the effectiveness of the model. To further increase the detection speed of the ChiliPCNN, its acceleration circuit is designed on the Xilinx Zynq7020 FPGA from the United States and optimized via fixed-point arithmetic and loop unrolling strategies. The optimized circuit reduces the latency to 5600 ns and consumes only 1.755 W of power, significantly improving the resource utilization rate and processing speed of the model. This system not only achieves rapid and accurate chili pepper variety and origin detection but also provides an efficient and reliable intelligent agricultural management solution, which is highly important for promoting the development of agricultural automation and intelligence.

## 1. Introduction

As modern agricultural technology has continued to advance, the chili pepper industry has witnessed unprecedented development opportunities. The diversity and market circulation rates of chili pepper varieties have significantly increased globally, providing consumers with a rich variety of choices. However, behind this prosperous scene, issues such as variety confusion and unknown origins have become increasingly prominent, constraining the healthy development of the industry and affecting consumer trust and food safety levels. Chili peppers, as seasoners with unique flavors and wide applications, include numerous varieties that range from spicy and pungent chilis to sweet and mildly spicy lantern peppers. Each variety has specific flavor characteristics and regional culture, serving as a key element in dish seasonings and culinary innovations. Moreover, the origin information of chili peppers is directly related to their quality, flavor characteristics, and ability to satisfy the stringent standards of specific geographical products; thus, this information is of immeasurable value for ensuring the authenticity of food and enhancing the competitiveness of markets.

Although some progress has been made regarding the identification of chili pepper varieties and their origins, many challenges remain. High-performance liquid chromatography (HPLC), which is a mature chemical composition analysis technique, can be used to accurately measure the contents of key components such as capsaicin and capsanthin in chili peppers, providing a scientific basis for performing variety identification [1]. However, this method requires high sample pretreatment standards, expensive instruments, and a relatively long analysis period, which is not conducive to quickly responding to market demands. Gas chromatography-mass spectrometry (GC-MS) provides origin tracing clues by separating and identifying the volatile compounds contained in chili peppers, but this approach is also limited by its complex sample processing procedures and high analysis costs [2]. Stable isotope ratio (SIR) analysis uses the natural distribution differences among element isotopes, along with geographical and environmental factors, to provide a new perspective for determining the origins of chili peppers [3]. However, this method is susceptible to environmental fluctuations and requires high-precision instruments. Molecular biology techniques, such as polymerase chain reaction (PCR) combined with sequencing, can achieve precise identification at the species level, but they have high technical thresholds, are costly, and impose requirements on sample storage conditions [4]. In addition, although sensory evaluation methods are intuitive and simple and rely on the experience of professional tasters, they are subjective, and ensuring objective consistency in the output results is difficult [5]. Therefore, the use of artificial intelligence technologies such as computer olfaction to assist in chili pepper identification is a major trend.

An electronic nose (e-nose), which is typically composed of a cross-sensitive gas sensor array with a pattern recognition algorithm unit, is considered an effective gas analysis method that can replace laboratory chemical and biological analysis methods. E-noses have been widely applied in fields such as environmental monitoring [6], medical diagnosis [7], and food analysis [8] and have particularly unique advantages and great potential in terms of detecting agricultural products in detail [9,10]. Liu et al. [11] employed a commercial PEN3 e-nose to detect volatile odors in chili peppers, successfully distinguishing between three types of chili peppers treated with different drying methods. Rasekh et al. [12] used a homemade e-nose in conjunction with various machine learning algorithms to identify sweet peppers and hot peppers. Yan et al. [13] utilized an e-nose to successfully identify five varieties of chili peppers and employed liquid chromatography to analyze their capsaicin content. Sun et al. [14] classified soybeans from six different origins via an e-nose system combined with adaptive deep learning models. The core of an e-nose lies in (1) its multivariate sensor array and (2) its sensor signal processing algorithm. The former can finely capture and effectively distinguish the specific characteristics of the volatile compounds released by various agricultural products, laying a solid foundation for highly precise identification and detection processes. The latter conducts feature extraction and pattern recognition on the response signals generated by the sensor arrays for various agricultural products, and it has been continuously optimized to improve its recognition accuracy. A significant direction concerning current e-nose technology research is the use of machine learning algorithms for pattern recognition. However, in practical applications, the low separability of signals and various interference factors encountered during the processes of data acquisition, processing and prediction severely affect the recognition accuracy of machine learning algorithms. With the development of deep learning, numerous research teams have explored its application potential in gas pattern recognition scenarios. For example, Feng et al. [15] developed a specialized domain node-level graph convolutional network (SDN-GCNet) paired with an e-nose to evaluate tea leaf quality levels across different harvest periods. Chen et al. [16] present a sensor-aware convolutional network (SACNet) that achieves precise chili pepper variety classification. Wang et al. [17] introduced BM-Net by leveraging a bidirectional mixing module (BMM) to identify volatile compounds effectively in Angelica dahurica. A model that adaptively fuses a lightweight transformer and an ELM was adopted by Sun et al. [18] to classify odors in refrigerators. The advanced CNN architectures proposed by Zhai et al. [19] and Yang et al. [20] demonstrated exceptional accuracy in industrial pollution gas recognition tasks. Compared with traditional machine learning methods, these deep learning frameworks not only attain improved recognition precision but also exhibit superior robustness and generalization capabilities when processing complex sensor data streams. However, most of the existing research on deep learning algorithms has focused on improving their gas recognition accuracy, neglecting the practical limitations of e-nose hardware implementations. Complex neural network models contain many parameters, imposing high performance demands on hardware, such as high-performance processors and large-capacity storage devices, which contradicts the detection capabilities pursued by portable e-noses and limits their application scope in instant detection scenarios.

Field-programmable gate arrays (FPGAs), with their highly customizable computing structures, parallel processing capabilities, excellent energy efficiency ratios, outstanding high-performance computing potential, and flexible reconfiguration characteristics, can achieve precise tuning to satisfy specific algorithmic requirements, thereby providing solutions with ultralow latency and ultrahigh throughput levels [21,22]. Therefore, FPGAs exhibit significant advantages when complex algorithms such as pattern recognition methods are deployed. For example, Luo et al. used an FPGA to accelerate CNNs for identifying plant diseases [23]. Neris et al. implemented efficient CNN operations on an FPGA to immediately process sensor data [24]. Zhang et al. propose an FPGA-based CNN accelerator that improves the speed and power efficiency of the CNN inference procedure by reducing the degree of data movement [25]. Research has demonstrated the potential of using FPGAs to accelerate pattern recognition applications. Traditional e-nose systems typically operate in isolation, where gas sensor arrays collect response data that are subsequently transmitted to a computer for analysis. Considering the real-time processing requirements, several studies have integrated odor recognition capabilities with FPGA platforms. For example, Ali et al. propose a principal component analysis (PCA)-based hardware–software codesign approach that combines e-nose technology with FPGA [26]. Mo et al. implemented a CNN deployed on FPGA for real-time detection of traditional Chinese medicines via e-nose data [27]. Tan et al. utilized FPGA as a hardware platform for industrial exhaust gas monitoring [28]. The integration of the e-nose and FPGA offers multiple advantages: (1) eliminating the need for external computers by executing recognition algorithms directly on the FPGA reduces system complexity and enhances portability, as new data can be processed immediately through the deployed FPGA model; (2) the FPGA’s parallel processing capabilities and reconfigurable logic enable efficient model acceleration with reduced inference latency; and (3) the FPGA’s low power consumption and cost efficiency make it suitable for embedded applications. These benefits highlight the growing importance of e-nose edge device design. However, current solutions predominantly rely on high-performance, resource-intensive FPGA platforms, creating insurmountable challenges for budget-constrained and power-sensitive e-nose applications. Moreover, the inherent limitations of these platforms often necessitate simplified pattern recognition algorithms that may achieve acceptable performance in specific scenarios but struggle with complex odor recognition tasks. Consequently, developing optimized design methodologies that enable high-precision, low-power, cost-effective, and rapid e-nose detection remains an unresolved challenge requiring innovative solutions.

In this study, we constructed a chili pepper variety and origin detection system. The system collects gases from thirteen different varieties of chili peppers and two specific chili peppers originating from seven regions via a gas sensor array to train and validate our proposed lightweight CNN, i.e., ChiliPCNN. This network integrates the advantages of CNNs in terms of their ability to co-learn features and efficiently rank raw input edges, as well as the merits of traditional methods such as multilayer perceptrons (MLPs), thereby forming an efficient and accurate classification model. Finally, the fully trained network model is deployed on an FPGA platform for forward inference purposes, realizing rapid and high-precision chili pepper variety and origin detection. The contributions of this work can be summarized as follows.

(1) We construct a system that integrates an FPGA and an e-nose for rapidly detecting the varieties and origins of chili peppers. The system can quickly and accurately identify thirteen chili pepper categories and effectively distinguish among seven different origins of two chili pepper varieties.

(2) We propose a hardware-friendly, lightweight ChiliPCNN model. This model is specifically designed for chili pepper data, enabling it to automatically mine deep features from raw sensor responses, effectively avoiding the feature extraction instability caused by differences in manual experience. By streamlining the hidden layer structure and eliminating bias terms, the ChiliPCNN significantly reduces the number of required parameters while ensuring that high detection accuracy is achieved, thereby reducing the storage requirements and computational burden imposed during the deployment process.

(3) We design and implement a ChiliPCNN acceleration circuit on the Xilinx Zynq7020 FPGA development board and comprehensively optimize the acceleration circuit by adopting fixed-point arithmetic technology and loop unrolling strategies. These optimization measures effectively improve the hardware resource utilization rate, reduce the power consumption level, and decrease processing delays.

## 2. Materials and Methods

### 2.1. System Architecture for Chili Pepper Variety and Origin Detection

**Figure 1:**
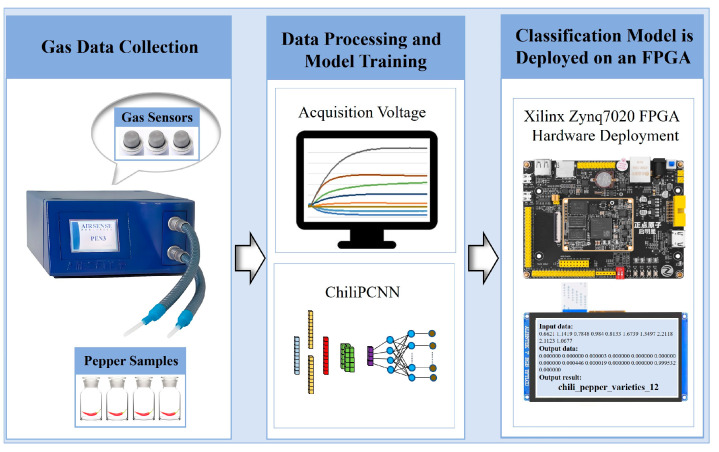
A pepper variety and origin detection system.

Figure 1 illustrates the system architecture constructed for chili pepper variety and origin detection, which integrates an FPGA with an e-nose. The core components include gas data acquisition, data processing, and model training mechanisms, as well as a forward inference module implemented on the FPGA. Gas data acquisition is performed by the AIRSENSE PEN3 e-nose from Germany, which incorporates a sensor array consisting of ten gas sensors with cross-selectivities, as listed in Table 1. This array is capable of effectively capturing and identifying various volatile components of chili pepper samples. After the sensor signals are amplified and digitized by the electronic circuitry, they are transmitted to a computer for further processing.

After the data undergo cleaning, labeling, and other processing tasks on the computer, they are used for model training and testing. The trained model is subsequently deployed on the Xilinx Zynq7020 FPGA development board (manufactured by Guangzhou Star Wing Electronic Technology Co., Ltd., Guangzhou, China). On the development board, Xilinx Zynq7020 (San Jose, CA, USA) serves as the core chip, which consists of a processing system (PS side) and a programmable logic module (PL side). The PS side includes a dual-core advanced RISC machine (ARM) Cortex-A9 CPU (manufactured by ARM Ltd., Cambridge, UK) operating at 1 GHz, which is primarily responsible for loading the data, displaying the results, and controlling the process. The PL side is utilized to design the hardware circuit for accelerating the ChiliPCNN model. When the FPGA development board receives data, it immediately displays the results on an RGB screen.

### 2.2. Sample Selection and Experimental Design

To ensure the scientific integrity of the experimental data, we established a collaborative partnership with the Chili Pepper Research Institute of the Guizhou Academy of Agricultural Sciences, Guiyang, China. Under rigorous expert guidance, three experimental groups were meticulously curated. Group 1 comprised 13 distinct chili pepper varieties (Qianjiao No. 8, Jiaoyang No. 1, Dafang zoujiao, Huaxi lajiao, Huangping xianjiao, Chuanjiao No. 19, Changla No. 7, Lafengguomei, Huiteng, Xiuting, Cuanjiao No. 1, Yanjiao 425, and Sanyingjiao No. 8) to evaluate the model’s performance in chili pepper variety identification. Groups 2 and 3 each contained Jiaoyang No. 6 and Xianjiao No. 301 varieties, respectively, sourced from seven geographical origins (Yunnan, Xinjiang, Chongqing, Hunan, Shaanxi, Neimenggu, and Henan) to assess the model’s accuracy in origin tracing. This design enabled the evaluation of model generalizability while mitigating potential biases arising from cultivar-specific origin differentiation effects.

All gas sensing analyses were performed via a commercial PEN3 e-nose under nondestructive conditions. During detection, gas molecules released from peppers interact with sensor arrays, inducing redox reactions that alter sensor conductivity. This change was quantified through the G/G_0_ ratio, where G represents the conductivity during chili pepper gas detection and G_0_ denotes the baseline conductivity measured with activated carbon-filtered gas. This methodology enables precise gas component analysis, providing reliable data for subsequent model validation.

The experiments were conducted in a controlled environment (26 ± 0.5 °C, 70 ± 10% RH) following the following procedures:

(1) The e-nose was preheated for 30 min to stabilize the signals before testing.

(2) Precisely weighed chili pepper samples (1.00 ± 0.05 g) were placed into precleaned sample bottles and sealed with airtight pads. To minimize random errors, 10 replicate sample bottles were prepared per sample.

(3) Prior to each measurement, the dynamic headspace method was performed by purging the system at 150 mL/min for 120 s to eliminate residual gases. Sample responses were then recorded at a 100 mL/min flow rate and a 1 Hz sampling frequency for 120 s. This protocol was repeated sequentially for all 10 sample bottles to complete one experimental cycle, which was replicated 10 times.

(4) Three detection protocols were implemented. The first group was designed to analyze the identification of 13 varieties of chili peppers. The second and third groups were designed to analyze the identification of the seven places of origin of the two chili peppers. In steps (2) to (3), three datasets were generated. The first group of detection experiments yielded a dataset comprising 13 varieties × 10 samples × 10 repetitions = 1300 data points, named Dataset A. Similarly, Datasets B and C were generated from the second and third groups of detection experiments, each consisting of 7 varieties × 10 samples × 10 repetitions = 700 data points. The raw sensor response curves from the e-nose measurements are presented in Figures S1–S3 in the Supplementary Materials.

### 2.3. ChiliPCNN Model for Chili Pepper Variety and Origin Detection

**Figure 2:**
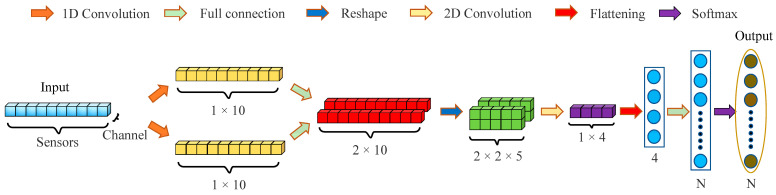
The structure of the ChiliPCNN.

To efficiently deploy a CNN on mobile devices for rapidly detecting the varieties and origins of chili peppers, we focus on two core design principles: (1) ensuring high detection accuracy and (2) maintaining low model complexity to ensure the compatibility of the model with conventional FPGAs in terms of their computational and storage requirements. On the basis of these principles, we conceive the lightweight ChiliPCNN architecture, which is specifically designed for chili pepper data, and its structure is detailed in Figure 2. The proposed model employs a multilevel feature fusion architecture to enable efficient feature learning. First, during the local feature extraction phase, parallel 1 × 3 and 1 × 5 multiscale convolutional kernels are utilized to capture short-range and medium-range spatial patterns, respectively. A global receptive field is subsequently constructed through fully connected layers to establish long-range dependencies among features and extract high-level abstract representations. The multilevel receptive fields are then superimposed to achieve parallel multiscale fusion. In the feature reorganization stage, the global feature vector is reconstructed into a 2D feature map through spatial–dimensional reorganization operations, preserving the spatial structure for subsequent convolution operations. Fine-grained local features are re-extracted from the reorganized features via convolutional kernels, enabling deep fusion of global contextual information with local detailed features. Finally, the fused features are mapped to a low-dimensional discriminative space through a feature projection layer, and a fully connected classifier is employed to accomplish the chili pepper variety and origin detection tasks. This architecture establishes a hierarchical feature learning pathway through an alternating local–global–local feature processing mechanism. Notably, we omit pooling layers and bias terms from both the convolutional and fully connected layers in our network model design. Given the limited nature of the input features, pooling would lead to information losses, severely impacting the resulting performance. Moreover, experiments show that the biases exhibited by the convolutional and fully connected layers contribute minimally to the results. Therefore, to simplify the model and facilitate its hardware implementation, we do not consider these layers in our design.

### 2.4. ChiliPCNN Acceleration Circuit

#### 2.4.1. ChiliPCNN Circuit Design

The acceleration circuit for the ChiliPCNN is initially described at a high level of abstraction via C/C++ with hardware-specific optimizations (pragmas) and synthesized into a hardware intellectual property (IP) core via high-level synthesis (HLS) [29]. Unlike direct implementation in hardware description languages (HDLs), HLS allows developers to focus primarily on algorithmic behavior via C/C++-like syntax, abstracting away the manual specification of low-level circuit elements (e.g., gates, registers, and exact timing). This significantly improves the initial development efficiency. The synthesized IP core is then integrated and optimized within the full FPGA system via Verilog HDL to ensure robust implementation, meet timing constraints, and avoid hazards.

The ChiliPCNN accelerator employs a hierarchical modular architecture at the HLS design level, where each functional layer (e.g., convolution, fully connected, and activation) is implemented as an independent HLS module. This strategy is driven by four key advantages: (1) decomposing the network into discrete HLS modules reduces algorithmic complexity, enabling independent development, verification, and optimization of each layer; (2) promoting the reuse of preoptimized HLS components across designs enhances flexibility and scalability; (3) hierarchical modularity facilitates the implementation of specific optimizations for each module, maximizing the hardware resource utilization rate and attaining improved performance. For example, the memory access process in the convolutional layers can be optimized to reduce latency, whereas the matrix multiplication operation executed in the fully connected layers can be optimized to achieve improved computational efficiency, significantly enhancing the overall performance and efficiency of the network. and (4) streamlining hardware mapping through modular HLS verification and subsequent system-level HDL implementation ensures reliability. The pseudocode structures for these core HLS modules are detailed in Algorithms S1–S4 in the Supplementary Materials.

**Figure 3:**
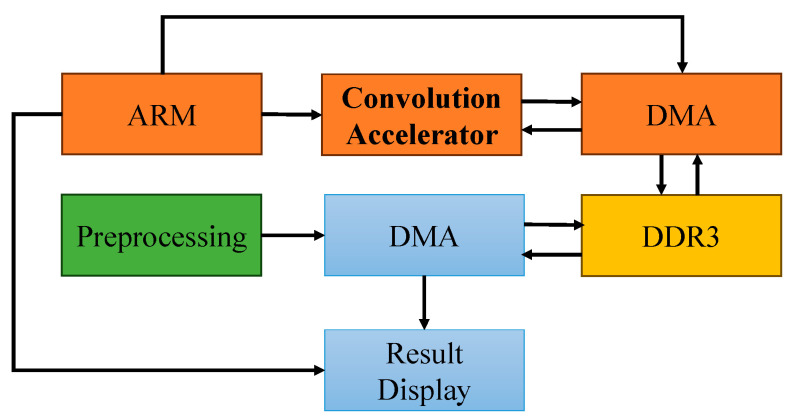
E-nose acceleration engine deployed to the FPGA.

Figure 3 shows the hardware architecture of the convolutional accelerator. This architecture employs an ARM processor as the primary control unit, coordinating multimodule operations through the advanced extensible interface (AXI) bus interface. Preprocessed input data are injected into the acceleration unit via the direct memory access (DMA) controller operating in the streaming mode, leveraging a parallel bus structure to minimize the latency induced during the data transfer step. Following the computation, the generated feature map data are retransmitted to the main memory through DMA channels, establishing a closed-loop processing pipeline that integrates “preprocessing → memory mapping → accelerated computation → result feedback” operations.

**Figure 4:**
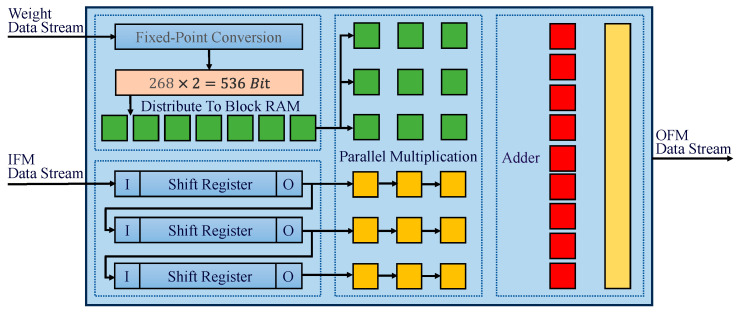
Convolution acceleration process.

The developed convolution acceleration process, visualized in Figure 4, centers on parallel computation principles. Specifically, convolution kernels and corresponding feature map segments are strategically mapped to multiple digital signal processing units (DSPs) to execute concurrent multiply accumulate operations. Ensuring precise alignment between the kernel parameters and feature data streams is critical, as kernels must operate on specific receptive fields within the feature map. The feature data transmitted via the AXI-Stream protocol undergo shift-register buffering in the FPGA to form convolutional windows, which are then multiplied in parallel with the weight matrices stored in block random access memory (BRAM). The accumulation of these parallel products yields the final convolution results, demonstrating the effective utilization of hardware parallelism while maintaining high computational accuracy through synchronized data orchestration.

**Figure 5:**
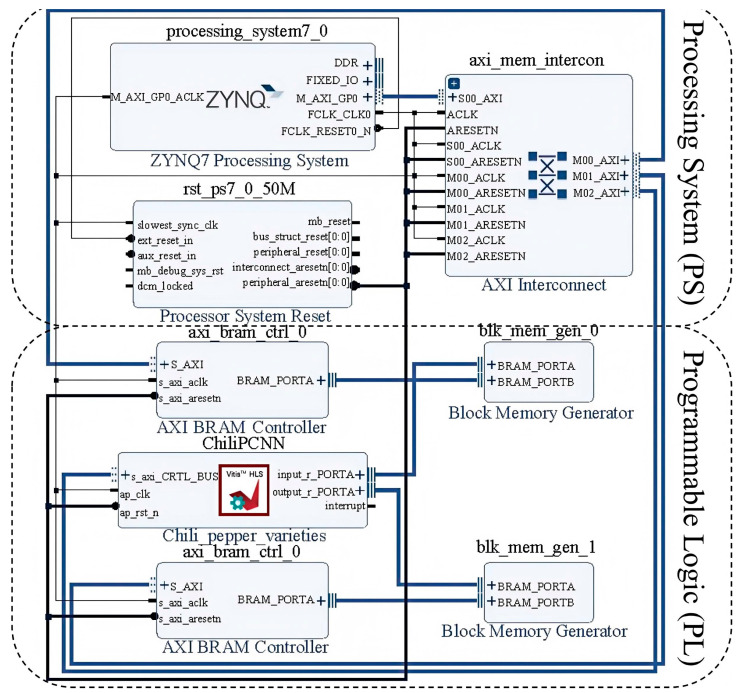
Circuit architecture of the e-nose.

Inspired by heterogeneous computing architectures, the complete e-nose acceleration system proposed in this work is structured as shown in Figure 5—which was exported following the system architecture design and verification process in Xilinx Vivado 2020.2 software—adopting a collaborative design paradigm integrating the PS and PL. The PS domain comprises an ARM Cortex-A9 central processing unit, a DDR3 dynamic memory module, and a universal asynchronous receiver/transmitter (UART) serial interface, whereas the PL domain incorporates a hardware-accelerated ChiliPCNN-based convolutional engine, an on-chip dynamic random access memory (DRAM) buffer, a DSP unit, and an AXI DMA high-speed bus. Synergistic acceleration is achieved through heterogeneous collaboration between the ARM Cortex-A9 (manufactured by ARM Ltd., UK) processor and the XC7Z020 ZYNQ device (manufactured by Guangzhou Star Wing Electronic Technology Co., Ltd., China). During the system initialization phase, the ARM processor configures the hardware platform and loads raw gas sensor data from the DDR3 memory. The subsequent data exchange process between the PS and PL is facilitated by the AXI4 protocol, which transmits preprocessed gas feature matrices to the hardware acceleration modules in the PL. The ChiliPCNN engine executes edge feature extraction and pattern matching with hardware-level parallelism, with the computed results returned to the PS via the AXI bus before being visualized on external displays through UART controllers for real-time monitoring.

#### 2.4.2. ChiliPCNN Circuit Optimization Method

To increase the hardware resource utilization rate of the ChiliPCNN circuit, reduce the power consumption of the model, minimize processing delays, and enable the rapid detection of chili pepper varieties and origins, we adopt fixed-point arithmetic technology and loop unrolling strategies to optimize the ChiliPCNN circuit.

In the original ChiliPCNN circuit, floating-point numbers were used for computations, since both the trained model weights and the collected data were represented in a floating-point format. However, owing to the complexity of its computation units and substantial storage requirements, floating-point arithmetic often results in significant hardware resource and power consumption levels, becoming a bottleneck that constrains the performance of the developed circuit. To overcome this limitation, we decide to integrate fixed-point arithmetic technology into the ChiliPCNN circuit to replace floating-point arithmetic. The conversion between fixed-point and floating-point numbers can be performed according to Equations (1) and (2).

(1) $$X_{\mathrm{fixed}}=\left\lfloor X_{\mathrm{float}}\times 2^{b}+0.5\right\rfloor$$

(2) $$X_{\mathrm{float}}=\frac{X_{\mathrm{fixed}}}{2^{b}}$$

Equation (1) is used to scale and round a floating-point number *X*_float_ to a fixed-point number *X*_fixed_, where *b* represents the number of decimal places. Equation (2) achieves reverse scaling conversion from a fixed-point number to a floating-point number. Compared with floating-point arithmetic, fixed-point arithmetic significantly reduces the hardware resource utilization rate by simplifying the representations of numerical values. Specifically, floating-point arithmetic requires complex computational units such as floating-point adders and multipliers, which not only occupy many lookup tables (LUTs) and digital signal processing units (DSPs) but also consume considerable power. In contrast, fixed-point arithmetic can be implemented with basic adders and multipliers, resulting in a relatively straightforward hardware implementation that greatly reduces the complexity and power consumption of hardware. Additionally, fixed-point arithmetic offers clear advantages in terms of latency. Floating-point arithmetic involves a series of complex operations, such as alignment, rounding, and normalization, which all require more clock cycles to complete than other operations do. Fixed-point arithmetic, on the other hand, directly performs arithmetic operations and can produce results quickly within fewer clock cycles. Therefore, adopting fixed-point arithmetic technology in the HLS design process can effectively reduce the hardware resource and power consumption levels while ensuring a certain level of computational accuracy and significantly improving the execution efficiency of the utilized hardware, thereby optimizing the performance of the circuit.

**Figure 6:**
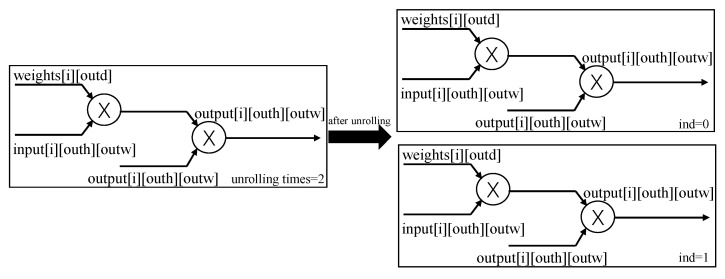
Unrolling process for a loop.

The ChiliPCNN circuit also contains numerous loop structures, such as the ‘for’ loops in its convolutional and fully connected layers. These loop structures often incur high control overhead and waiting times during the execution procedure, severely impacting the performance of the circuit. To address this issue, we employ a loop unrolling strategy. The basic idea of this strategy is to replicate the code within the loop body multiple times, allowing multiple loop iterations to be executed in parallel. This parallel processing approach not only significantly increases the hardware throughput level but also effectively reduces the induced branch prediction error rates. In the specific implementation of the ChiliPCNN circuit, we unroll the key loop structures. Taking the convolutional ‘for’ loop with a kernel size of five as an example, the loop requires five iterations, each of which includes a multiplication circuit and an addition circuit. Considering the limited number of available memory ports, we reasonably set the unrolling factor to two. The principle of loop unrolling is illustrated in Figure 6. After completing the unrolling step, two identical circuit modules are formed and complete their first two loop iterations in parallel, followed by one circuit module for completing the remaining iteration. Although the optimized circuit obtained after unrolling occupies twice the FPGA resources, it saves 2/5 of the execution time relative to the original version. Given sufficient FPGA hardware resources and other optimization measures, we can significantly improve the computational efficiency of the convolution operations by trading hardware resources for time. When performing chili pepper variety and origin detection tasks, the optimized circuit can produce detection results more quickly.

## 3. Results and Discussion

### 3.1. Experimental Setup

To facilitate model training and testing, each dataset is split into a training set and a test set at a 7:3 ratio, ensuring the independence and validity of the data. The experimental section is divided into two groups: (1) In the first group of experiments, each data sample contained in the training set is divided into independent training samples at 1 second intervals for model training. Similarly, each data sample in the test set is also divided according to the same principle to generate independent test samples, which are used to evaluate the performance of the model. In addition, we predict the test samples in a second-by-second manner starting from the first second according to the sampling time order and calculate the accuracy achieved for each second to assess the timeliness of the model comprehensively; (2) in the second group of experiments, considering that the chili gas may not fully contact the sensor array within the first 20 seconds, we select the portion from the 21st second to the 120th second of each data sample in the training set, dividing it into training instances at 1 second intervals for model training. The data samples in the test set are processed in the same way, i.e., the portion from the 21st section to the 120th second is divided into test instances. We also predict the test samples in a second-by-second manner starting from the 21st second according to the sampling time order and calculate the accuracy for each second starting from the 21st second to more accurately evaluate the performance of the model in the stable stage.

During the training process, we adopt the cross-entropy loss function as the loss metric for the model. To ensure the stability and efficiency of the training process, the batch size of the model is set to 32. To optimize the learning process of the model, the initial learning rate is set to 0.01, and after every 10 epochs, the learning rate is multiplied by 0.95 to gradually fine-tune the model parameters. The entire training process iterates 500 times to ensure that the model can fully learn the characteristics of the input data. In addition, we choose the stochastic gradient descent (SGD) optimizer to perform parameter optimization and weight updates, which drive the model closer to the optimal solution.

During the acceleration circuit design phase, preliminary experiments are conducted to evaluate various fixed-point optimization strategies. Through a comparative analysis, we establish that converting floating-point numbers to fixed-point representations with a 16-bit width is optimal. To prevent numerical overflow and minimize the quantization loss simultaneously, we preserve 12 fractional bits. This configuration results in an average inference accuracy reduction of merely 0.3% across the three datasets, maintaining the precision loss within an acceptable margin.

To verify the effectiveness and superiority of the proposed ChiliPCNN model, we select multiple lightweight machine learning and deep learning networks for comparative experiments, including a mixed-kernel and variable-dimensional memristive convolutional neural network (MixVMCNN) [30], an MLP [31], a single-layer perceptron (SLP) [32], a 1D CNN [33], a 2D CNN [34], a gated recurrent unit (GRU) [35], long short-term memory (LSTM) [36], a convolutional spiking neural network (CSNN) [37], and the classic MobileNetv2 network [38]. In the experiments, the ratio of the number of samples in the training set to that in the test set is maintained at 7:3, and all methods are repeated ten times to ensure the validity of the experimental results. To conduct a comprehensive performance assessment, we employ five widely used metrics: accuracy, precision, recall, specificity, and the F1 score. Their definitions are shown in Equations (3)–(7).

(3) $$\mathrm{accuracy}=\frac{\mathrm{TP}+\mathrm{TN}}{\mathrm{TP}+\mathrm{TN}+\mathrm{FP}+\mathrm{FN}}$$

(4) $$\mathrm{precision}=\frac{\mathrm{TP}}{\mathrm{TP}+\mathrm{FP}}$$

(5) $$\mathrm{recall}=\frac{\mathrm{TP}}{\mathrm{TP}+\mathrm{FN}}$$

(6) $$\mathrm{specificity}=\frac{\mathrm{TN}}{\mathrm{TN}+\mathrm{FP}}$$

(7) $$F1-\mathrm{score}=\frac{2\times \mathrm{precision}\times \mathrm{recall}}{\mathrm{precision}+\mathrm{recall}}$$

where TP represents the number of true positives, FP represents the number of false positives, TN represents the number of true negatives, and FN represents the number of false negatives. In addition, we report three important computational efficiency metrics: the number of parameters (#Params), the number of floating-point operations (#FLOPs), and the model size. #Params refers to the total number of trainable parameters contained in the model, which directly affects the storage requirements and computational overhead of the model. A smaller #Params can reduce the computational cost and memory usage of the model, improving its efficiency, especially for resource-constrained devices. The #FLOPs refers to the number of floating-point operations required during a single forward pass of the model, which is an important computational efficiency indicator. A smaller #FLOPs typically results in a faster inference speed. The model size is the amount of memory required to store the model, which depends on the total number of model parameters. These metrics assess the computational demands and the model architecture to demonstrate its suitability for use in resource-constrained environments.

We subsequently select the three directly deployable models with the highest accuracies on Zynq7020, namely, the MLP, 1D CNN, and 2D CNN, and implement their hardware circuits through the HLS design on Dataset A. We then conduct a detailed comparison with the optimized and accelerated ChiliPCNN circuit in terms of its hardware resource utilization, power consumption level, processing delay, and other indicators.

### 3.2. Chili Pepper Variety and Origin Detection Results of ChiliPCNN

**Figure 7:**
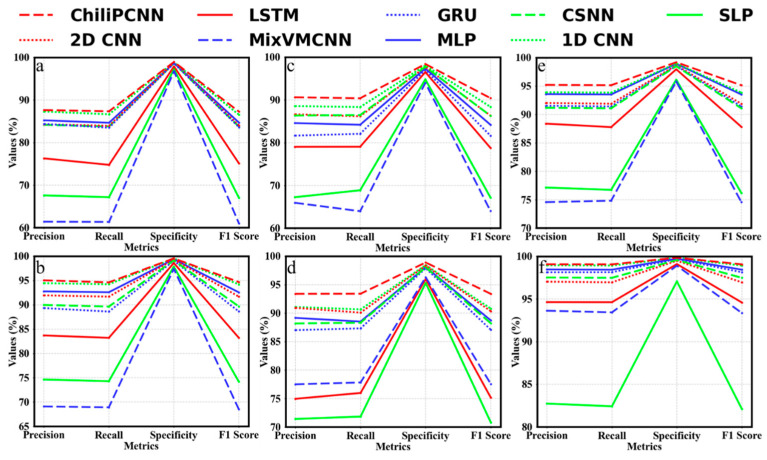
Comparison among the metrics produced across different models. (**a**,**b**): Dataset A; (**c**,**d**): Dataset B; (**e**,**f**): Dataset C. Each dataset shows two experimental configurations.

In this experiment, we conduct a comprehensive and detailed comparative analysis of the performance achieved by the ChiliPCNN model and multiple other models in chili pepper variety and origin detection tasks. The experimental results are presented in detail in Table 2, Table 3 and Table 4. The data clearly reveal that the ChiliPCNN algorithm has superior test accuracy in both sets of experiments designed for the three different datasets. Notably, while the conventional MobileNetv2 architecture exhibits marginally superior accuracy, its deployment feasibility is significantly compromised by its prohibitive #Params and #FLOPs. Therefore, it is necessary to compare the ChiliPCNN with other models at comparable scales. Specifically, on Dataset A, ChiliPCNN significantly outperforms the other algorithms in terms of the test accuracies achieved in both sets of experiments; compared with the third-best 1D CNN, its accuracies are approximately 0.71% and 0.39% higher, respectively, and compared with the worst-performing MixVMCNN, its advantages are even more significant, with accuracy improvements of approximately 26.01% and 25.7%, respectively. On Dataset B, the ChiliPCNN also performs excellently. In both sets of experiments, its test accuracies rank second, approximately 0.91% and 1.84% higher than those of the third-best 2D CNN. In the first set of experiments, its accuracy is approximately 26.43% higher than that of the worst-performing MixVMCNN, and in the second set of experiments, the accuracy is approximately 21.58% higher than that of the worst-performing SLP network. Furthermore, on Dataset C, which contains chili pepper volatiles with richer compound diversity that provides more discriminative information for model training and significantly enhances the feature representation ability of the model, ChiliPCNN continues to maintain its leading position. In the second set of experiments, our proposed model achieves 0.1% higher accuracy than MobileNetv2 does. Compared with the immediately following 1D CNN models, it yields accuracy improvements of approximately 1.63% and 0.14%. Compared with those of the worst-performing networks, the accuracies of the approach are approximately 20.34% greater than those of the MixVMCNN in the first set of experiments and approximately 16.65% greater than those of SLP in the second set of experiments. This can be intuitively seen from the line graph shown in Figure 7, where a and b represent the two sets of experiments conducted on Dataset A, c and d represent the two sets of experiments conducted on Dataset B, and e and f represent the two sets of experiments conducted on Dataset C. In each set of experiments conducted across the three different datasets, the ChiliPCNN model achieves the highest values for the four evaluation metrics: precision, recall, specificity, and F1 score. This more comprehensively reflects the excellent performance of the ChiliPCNN model in chili pepper variety and origin detection tasks.

A further analysis of the data contained in Table 2, Table 3 and Table 4 reveals that the ChiliPCNN model has very small #Params, #FLOPs, and model size values (in Bytes). In terms of its hardware circuit design, the model requires significantly fewer resources, such as BRAM, DSPs, and LUTs, making it easily deployable on lower-performance FPGAs and satisfying the hardware resource requirements of most FPGAs. For chili pepper variety identification, the model has only 268 parameters and requires only 364 FLOPs per second. For chili pepper origin tracing, the model has only 244 parameters and requires only 340 FLOPs per second. Although the MixVMCNN model has the fewest #Params and the SLP model has the fewest #FLOPs, their detection accuracies are low, and their feature extraction capabilities are weak. On the other hand, the 1D CNN model and 2D CNN model, which offer good detection accuracy, have #FLOPs that are multiple times greater than those of the ChiliPCNN, and they also require significantly more hardware resources.

In summary, for both chili pepper variety identification and origin tracing, the ChiliPCNN model achieves the highest test accuracy in two rounds of experiments conducted across three datasets. Additionally, it consumes very few hardware resources, making it more suitable for deployment on FPGAs. It significantly outperforms all the other compared algorithms.

### 3.3. Results of Rapid Detection Experiments

**Figure 8:**
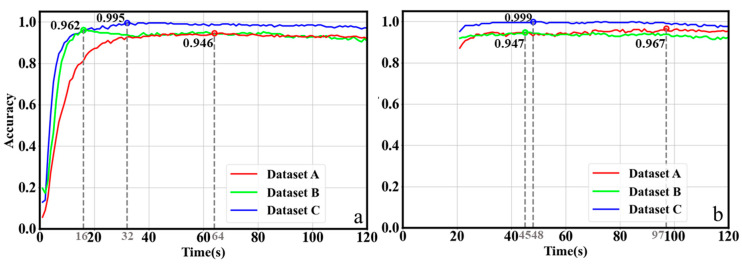
Second-by-second accuracy. (**a**,**b**) compare the experimental results of the first and second groups, respectively.

We conduct two rounds of experiments on the proposed ChiliPCNN model across three datasets, predicting test samples on a second-by-second basis and calculating the accuracy achieved for each second. The results are shown in Figure 8. In the first twenty seconds of the first round of the experiments, the accuracy of the model is relatively low. However, in the second round of experiments, starting from the 21st second, the accuracy attained for each second exceeds 87%, with a more stable performance. Therefore, in practical applications, we choose the model trained in the second round of experiments. A further analysis of the results derived from the second round of experiments reveals the following: at the 97th second, the accuracy achieved for the thirteen-class chili pepper variety identification task reaches a peak for the first time, at 96.7%; at the 45th second, the accuracy attained for the seven-origin tracing task performed on Dataset B reaches a peak for the first time, at 94.7%; and at the 48th second, the accuracy achieved for the seven-origin tracing task conducted on Dataset C reaches a very high value of 99.9%. The variety identification and original tracing tasks require the complete collection of 120 s of gas data. However, on the basis of the results of this experiment, we can set the gas collection time to 97 s, 45 s, and 48 s for different tasks. Once these set times are reached, detection results can be obtained, thereby achieving the goal of rapid detection.

### 3.4. Optimization Results of the ChiliPCNN Acceleration Circuit

To effectively deploy the ChiliPCNN model on a resource-constrained FPGA and accelerate the process of detecting chili pepper varieties and origins, we design and implement the ChiliPCNN acceleration circuit. To further enhance the performance of the circuit, we optimize it via fixed-point arithmetic technology and loop unrolling strategies. Table 5 provides a detailed comparison between the versions of the ChiliPCNN model before and after implementing the optimization process, along with the MLP, 1D CNN, and 2D CNN models, in terms of their latency, resource utilization rates, and accuracy. The unoptimized ChiliPCNN circuit has a latency of 8800 ns and consumes 42 BRAM modules, 57 DSPs, 10,878 flip flops (FFs), and 13,057 LUTs, accounting for 30%, 25.91%, 10.22%, and 24.54% of the total FPGA resources, respectively. After the optimization step, the circuit latency is reduced to 5600 ns, representing a speedup of 36.36%. Simultaneously, the resource consumption level is effectively controlled, with 38 BRAMs, 83 DSPs, 5633 FFs, and 8833 LUTs, accounting for 27.14%, 37.73%, 5.29%, and 16.70% of the total FPGA resources, respectively. Despite a slight decrease of 0.37% in the accuracy of the model, the optimized version demonstrates superior performance in terms of latency and most resource metrics. A key advantage of the optimized model lies in its heightened DSP efficiency, which directly contributes to reduced processing delays and logical resource conservation, creating an operational headroom for subsequent system enhancements. Compared with the other models, the optimized ChiliPCNN circuit has the best performance in terms of latency, and it has the lowest resource utilization rate with respect to its FFs and LUTs. Although the resource utilization level of the BRAM is slightly higher than those of the MLP and 1D CNN, this is primarily due to the network structure of the ChiliPCNN model, which requires more memory buffering to store the intermediate results. This architectural requirement, however, is compensated by the demonstrated superiority of the model in terms of accuracy, surpassing the comparative benchmarks.

Furthermore, device power consumption is an essential indicator for evaluating performance. According to the data shown in Table 6, the total power consumption of the optimized ChiliPCNN circuit deployed on the FPGA is 1.755 W, which is lower than that observed before implementing the optimization scheme and those of the other models. The power dissipation of PS7 mainly originates from the ARM processor and its peripheral modules, whereas the core power consumption of acceleration circuits focuses on the clock network and block storage modules. Specifically, the energy consumption of the BRAM not only includes static retention power during data storage but also generates significant dynamic switching power during frequent reading/writing operations. Therefore, optimizing the data transmission mechanism to reduce the dynamic power consumption level is crucial for improving the overall energy efficiency of the model.

To systematically evaluate the effectiveness of different ChiliPCNN circuit optimization strategies, ablation experiments are employed in this study by incrementally introducing fixed-point arithmetic and loop unrolling techniques to quantitatively analyze the performance contributions of each optimization strategy. According to the data shown in Table 7, after implementing fixed-point arithmetic, the circuit latency is significantly reduced by 30.45% to 6120 ns, which is accompanied by synergistic reductions across multiple hardware resources. This validates the dual benefits of numerical precision optimization for enhancing the computational efficiency and controlling the hardware complexity of the model. The subsequent integration of loop unrolling technology through the strategic allocation of BRAM and DSP resources yields an additional 8.49% latency reduction to 5600 ns at the cost of increased memory and processing unit utilization. This optimization approach effectively strengthens the real-time performance advantages of FPGAs in edge computing scenarios. In summary, the optimized ChiliPCNN circuit excels in terms of latency, resource utilization, and power consumption, providing strong support for the rapid and efficient deployment of the chili pepper variety and origin detection.

To evaluate ChiliPCNN’s hardware deployment efficiency in practical edge scenarios (real-time chili pepper recognition), we compared the inference speed of ChiliPCNN across different hardware platforms (see table notes for computational device details), and the comparison results are given in Table 8. The inference speeds were averaged across multiple tests. The results show that the FPGA achieves single-chili recognition in 7071 ns, significantly outperforming the CPU (128,800 ns) with an 18.21 × speedup. Even compared with the GPU’s parallel batch processing (32 samples per batch), the FPGA remains 3529 ns faster for single-sample inference. While increasing the GPU’s batch size improves its throughput, the FPGA development board (including peripheral components) has substantially lower costs than CPU/GPU solutions that rely solely on discrete chips. Given that practical applications typically require single-inference results, the FPGA’s fixed-point arithmetic optimizes resource utilization and reduces latency, whereas its pipeline architecture enables parallel computing—providing critical advantages in resource-constrained scenarios.

## 4. Conclusions

A lightweight network, ChiliPCNN, is presented in this paper for the rapid identification of chili pepper varieties and their origins. The ChiliPCNN model is meticulously crafted, with only 268 parameters and 364 FLOPs required for the variety identification task and even fewer parameters (244) and FLOPs (340) needed for the origin tracing task. This design makes ChiliPCNN highly suitable for deployments and applications in resource-constrained embedded systems. To address the practical needs of chili pepper variety and origin identification tasks, we integrate e-nose technology with a gas sensor array to capture the volatile compound features of chili samples, which serve as input data for training the ChiliPCNN model. To further increase the inference speed of the ChiliPCNN, we design an FPGA-based ChiliPCNN accelerator. This accelerator was developed via HLS tools, enabling efficient hardware implementation of the ChiliPCNN model. During the accelerator design process, we employ various optimization strategies, such as fixed-point arithmetic and loop unrolling, to fully leverage the performance of the acceleration circuit. These optimization techniques not only effectively improve the processing speed and resource utilization rate of the acceleration circuit of the model but also significantly reduce its power consumption. After the optimization step, the latency of the acceleration circuit is reduced to 5600 ns, and the power consumption level is decreased to 1.755 W. Ultimately, a comprehensive e-nose acceleration circuit is designed, enabling the transmission of acquired data to the FPGA for forward inference processing. The computed results are then delivered to the display screen in real time for continuous monitoring.

In summary, the chili pepper variety and origin detection system proposed in this paper, which integrates an FPGA and e-nose technology, achieves a good balance between high accuracy and rapidity in chili pepper variety and origin identification scenarios. This system provides an efficient and reliable solution for the intelligent management of the chili industry. Moreover, this study offers a promising reference example for the use of artificial intelligence technology in agricultural product quality detection situations within the agricultural sector, which is highly important for promoting the development of agricultural automation and intelligence. Notably, this study also has certain limitations, which provide directions for future work: (1) while the current algorithm performs excellently under standard laboratory conditions, future research could further enhance its robustness against complex environmental factors to ensure system stability in broader practical application scenarios; and (2) the FPGA-based hardware acceleration scheme effectively guarantees processing speed; however, future efforts could focus on more refined optimization of FPGA implementation in terms of power consumption and resource utilization to increase the system’s energy efficiency ratio and potential deployment flexibility.

## Figures and Tables

**Table 1:** Sensors contained within the e-nose.

Sensor	Sensitive Gas
W1C	Aromatic compounds
W5S	Hydrocarbon compounds
W3C	Ammonia
W6S	Hydrogen
W5C	Aromatic hydrocarbons
W1S	Methane
W1W	Sulfides
W2S	Ethanol
W2W	Organic sulfides
W3S	Alkanes

**Table 2:** Performance comparison (%) among various models on Dataset A.

	Metric	MLP	SLP	1D CNN	2D CNN	GRU	LSTM	CSNN	MixVMCNN	MobileNetv2	ChiliPCNN
120 s	Accuracy	84.19	68.86	88.34	89.48	82.07	79.05	86.45	63.96	91.82	90.39
	Precision	84.58	67.24	88.56	86.64	81.65	79.03	86.36	65.93	93.00	90.62
	Recall	84.19	68.86	88.34	86.17	82.07	79.05	86.45	63.96	91.82	90.39
	Specificity	97.36	94.81	98.06	97.70	97.01	96.51	97.74	93.99	98.64	98.40
	F1 score	84.18	67.14	88.38	86.32	81.62	78.76	86.29	64.00	91.97	90.42
100 s	Accuracy	88.52	71.83	90.66	91.57	87.32	75.98	88.32	77.82	94.44	93.41
	Precision	89.17	71.41	91.07	90.93	87.02	74.95	88.19	77.50	94.78	93.39
	Recall	88.52	71.83	90.66	90.09	87.32	75.98	88.32	77.82	94.44	93.41
	Specificity	98.09	95.31	98.44	98.35	97.89	96.00	98.05	96.30	99.07	98.90
	F1 score	88.73	70.80	90.79	90.34	87.08	75.15	88.22	77.56	94.48	93.39
	#Params	295	77	275	291	297	291	277	44	2,232,295	244
	#FLOPs	272	70	928	5425	325	316	582	140	2,375,472	340
	Model size	1180	308	1100	1164	1188	1164	1108	176	8,929,180	976

**Table 3:** Performance comparison (%) among various models on Dataset B.

	Metric	MLP	SLP	1D CNN	2D CNN	GRU	LSTM	CSNN	MixVMCNN	MobileNetv2	ChiliPCNN
120 s	Accuracy	84.65	67.16	86.64	85.45	83.49	74.76	83.79	61.34	88.37	87.35
	Precision	85.23	67.58	87.30	84.29	84.38	76.28	84.17	61.40	89.20	87.63
	Recall	84.65	67.16	86.64	83.94	83.49	74.76	83.79	61.34	88.37	87.35
	Specificity	98.72	97.26	98.89	98.79	98.62	97.90	98.65	96.78	99.03	98.95
	F1 score	84.69	66.99	86.53	83.99	83.59	75.09	83.75	60.97	88.39	87.26
100 s	Accuracy	92.59	74.29	94.23	93.45	88.61	83.21	89.64	68.92	95.73	94.62
	Precision	92.76	74.64	94.46	91.95	89.33	83.71	89.97	69.10	95.95	95.04
	Recall	92.59	74.29	94.23	91.68	88.61	83.21	89.64	68.92	95.73	94.62
	Specificity	99.38	97.86	99.52	99.31	99.05	98.60	99.14	97.41	99.64	99.55
	F1 score	92.57	74.20	94.22	91.69	88.64	83.23	89.68	68.53	95.72	94.69
	#Params	397	143	377	321	333	321	319	68	2,239,981	268
	#FLOPs	368	130	1024	5448	355	340	618	164	2,383,152	364
	Model size	1588	572	1508	1284	1332	1284	1276	272	8,959,924	1072

**Table 4:** Performance comparison (%) among various models on Dataset C.

	Metric	MLP	SLP	1D CNN	2D CNN	GRU	LSTM	CSNN	MixVMCNN	MobileNetv2	ChiliPCNN
120 s	Accuracy	93.52	76.73	93.85	92.37	91.42	87.77	91.09	74.81	95.82	95.15
	Precision	93.58	77.13	93.90	92.02	91.53	88.38	91.18	74.56	96.03	95.21
	Recall	93.52	76.72	93.85	91.89	91.42	87.77	91.09	74.81	95.82	95.15
	Specificity	98.92	96.12	98.97	98.65	98.57	97.96	98.51	95.80	99.30	99.19
	F1 score	93.52	76.16	93.84	91.88	91.40	87.82	91.09	74.56	95.86	95.14
100 s	Accuracy	98.44	82.42	98.93	98.03	98.15	94.63	97.50	93.44	98.97	99.07
	Precision	98.47	82.74	98.97	97.04	98.14	94.64	97.53	93.63	99.00	99.08
	Recall	98.44	82.42	98.93	96.96	98.15	94.63	97.49	93.44	98.97	99.07
	Specificity	99.74	97.07	99.82	99.49	99.69	99.10	99.58	98.91	99.83	99.85
	F1 score	98.44	82.10	98.93	96.96	98.14	94.59	97.49	93.36	98.97	99.07
	#Params	295	77	275	291	297	291	277	44	2,232,295	244
	#FLOPs	272	70	928	5425	325	316	582	140	2,375,472	340
	Model size	1180	308	1100	1164	1188	1164	1108	176	8,929,180	976

**Table 5:** Latency and resource consumption levels of different models.

Models	Latency	BRAM/%	DSP/%	FF/%	LUT/%	Accuracy
MLP	5800 ns	12/8.57%	89/40.45%	10,371/9.75%	15,389/28.93%	84.19%
1D CNN	6960 ns	36/25.71%	89/40.45%	15,522/14.59%	18,585/34.93%	88.34%
2D CNN	44,860 ns	46/32.86%	87/39.54%	19,815/18.62%	25,378/47.70%	89.48%
ChiliPCNN	8800 ns	42/30%	57/25.91%	10,878/10.22%	13,057/24.54%	90.39%
Optimized	5600 ns	38/27.14%	83/37.73%	5633/5.29%	8883/16.70%	90.02%

Note: the total resources of the Zynq7020 development board include 140 BRAMs, 220 DSPs, 106,400 FFs, and 53,200 LUTs.

**Table 6:** Power consumption (W) comparison among different models.

Metric	Optimized	ChiliPCNN	MLP	1D CNN	2D CNN
Dynamic	1.611	1.639	1.642	1.694	1.720
Clocks	0.009	0.018	0.024	0.026	0.031
Signals	0.021	0.033	0.035	0.061	0.071
Logic	0.017	0.027	0.029	0.036	0.042
BRAM	0.007	0.014	0.003	0.014	0.016
DSP	0.026	0.016	0.020	0.027	0.029
PS7	1.531	1.531	1.531	1.531	1.531
Static	0.144	0.145	0.143	0.146	0.148
Total	1.755	1.784	1.785	1.840	1.868

**Table 7:** Results of an ablation study concerning the ChiliPCNN circuit optimization strategy.

Method	Latency (ns)	BRAM	DSP	FF	LUT
Unoptimized	8800	42	57	10,878	13,057
Fixed-point	6120	18	53	5243	7670
Fully optimized	5600	38	83	5633	8883

**Table 8:** Effect of the ChiliPCNN implementation on different devices.

Platform	Batch Size	Average Time (ns)	Speedup
CPU	-	128,800	1.0×
CPU + GPU	1	354,000	0.36×
CPU + GPU	32	10,600	12.15×
CPU + GPU	64	5900	21.83×
FPGA	-	7071	18.21×

Note: CPU: AMD Ryzen 9 7945HX (manufactured by AMD, Santa Clara, CA, USA), GPU: NVIDIA GeForce RTX 4060 (manufactured by NVIDIA, San Jose, CA, USA), FPGA: Xilinx Zynq7020 (manufactured by Xilinx, San Jose, CA, USA).

## Data Availability

The original contributions presented in the study are included in the article/Supplementary Materials, further inquiries can be directed to the corresponding author.

## Supplementary Materials

The following supporting information can be downloaded at https://www.mdpi.com/article/10.3390/foods14152612/s1. Figure S1: The original data curves of the e-nose for Dataset A. The subplots (a–m) correspond to the original curves of the sensor responses to the 13 varieties of chili peppers (Qianjiao No. 8, Jiaoyang No. 1, Dafang zoujiao, Huaxi lajiao, Huangping xianjiao, Chuanjiao No. 19, Changla No. 7, Lafengguomei, Huiteng, Xiuting, Cuanjiao No. 1, Yanjiao 425, and Sanyingjiao No. 8); Figure S2: The original data curves of the e-nose for Dataset B. The subplots (a–g) correspond to the original curves of the sensor responses to the 7 origins of the chili peppers (Yunnan, Xinjiang, Chongqing, Hunan, Shaanxi, Neimenggu, and Henan); Figure S3: The original data curves of the e-nose for Dataset C. The subplots (a–g) correspond to the original curves of the sensor responses to the origins of the chili peppers (Yunnan, Xinjiang, Chongqing, Hunan, Shaanxi, Neimenggu, and Henan); Algorithm S1: HLS module of Conv1D layer; Algorithm S2: HLS module of Conv2D layer; Algorithm S3: HLS module of fully connected layer; Algorithm S4: HLS module of Softmax.
