# Supplementary material for: An Integrated Lightweight Neural Network Design and FPGA-Accelerated Edge Computing for Chili Pepper Variety and Origin Identification via an E-Nose
Source: Foods. 2025 Jul 25;14(15):2612. doi: 10.3390/foods14152612 (PMC12346674; doi:10.3390/foods14152612)
Supplement: Supplementary file 1 [file foods-14-02612-s001.zip › foods-3698313-supplementary.pdf]

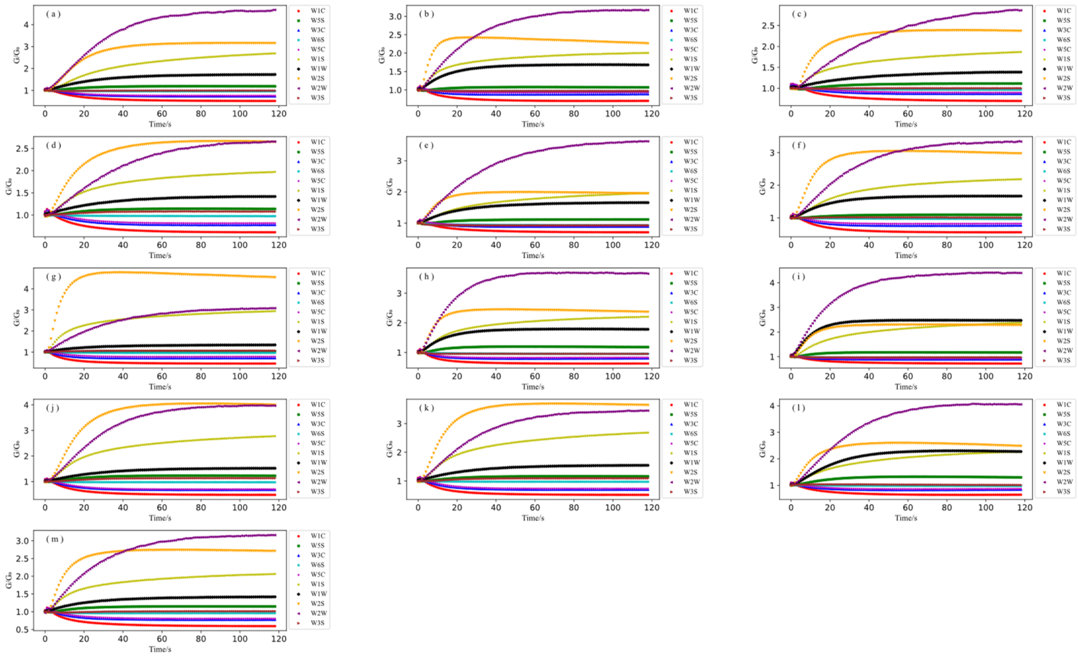

Figure S1. The original data curves of the e-nose for Dataset A. The subplots (a-m) correspond to the original curves of the sensor responses to the 13 varieties of chili peppers (Qianjiao No. 8, Jiaoyang No. 1, Dafang zoujiao, Huaxi lajiao, Huangping xianjiao, Chuanjiao No. 19, Changla No. 7, Lafengguomei, Huiteng, Xiuting, Cuanjiao No. 1, Yanjiao 425, and Sanyingjiao No. 8).

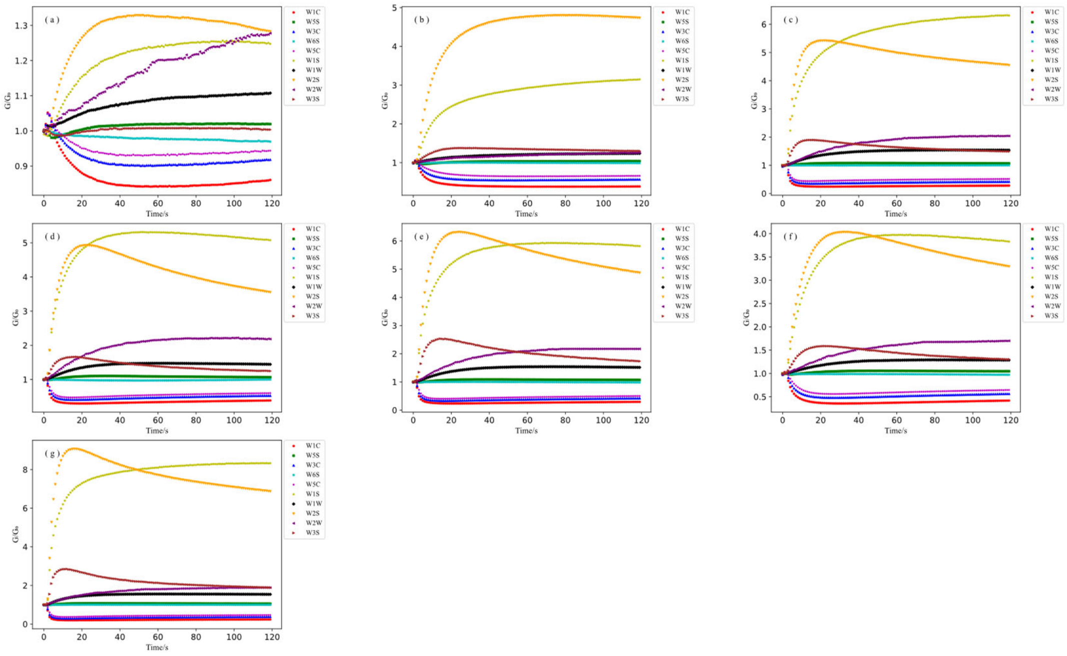

Figure S2. The original data curves of the e-nose for Dataset B. The subplots (a-g) correspond to the original curves of the sensor responses to the 7 origins of the chili peppers (Yunnan, Xinjiang, Chongqing, Hunan, Shaanxi, Neimenggu, and Henan).

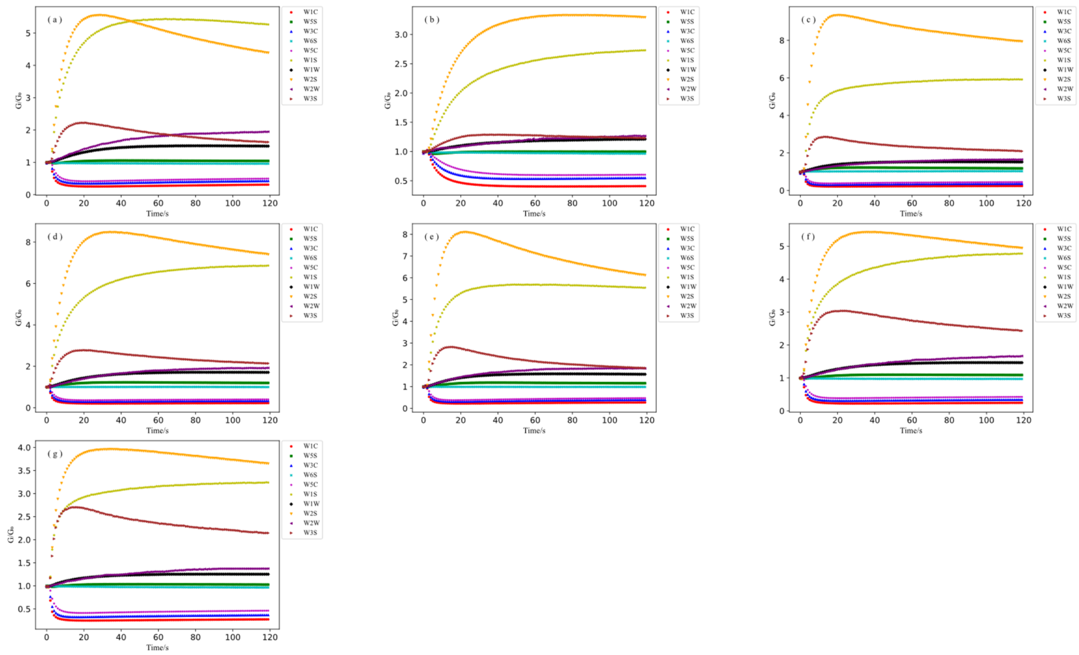

Figure S3. The original data curves of the e-nose for Dataset C. The subplots (a-g) correspond to the original curves of the sensor responses to the origins of the chili peppers (Yunnan, Xinjiang, Chongqing, Hunan, Shaanxi, Neimenggu, and Henan).

Algorithm S1: HLS module of Conv1D layer

```
Input: input sequence
       convolution kernel
       padding size
Output: output sequence
for i = 0 to input_size − 1 do
    sum = 0
    for j = 0 to kernel_size − 1 do
        idx = i + j − padding
        if idx ⩾ 0 and idx < input_size then
            sum = sum + input[idx] × kernel[j]
        end if
    end for
    output[i] = sum
end for
Return output sequence
```

Algorithm S2: HLS module of Conv2D layer

```
Input: input tensor
       convolution kernel
Output: output feature map
for oh = 0 to f − 1 do
    for ow = 0 to e − 1 do
        sum = 0
        for ic = 0 to h − 1 do
            for kh = 0 to i − 1 do
                for kw = 0 to j − 1 do
                    ih = oh + kh
                    iw = ow + kw
                    if ih < b and iw < c then
                        sum = sum + input[ic][ih][iw] × kernel[ic][kh][kw]
                    end if
                end for
            end for
        end for
        output[0][oh][ow] = sum
    end for
end for
Return output feature map
```

Algorithm S3: HLS module of Fully connected layer

Input: input vector
    weight matrix
Output: output vector
for i = 0 to output_size − 1 do
    sum = 0
    for j = 0 to input_size − 1 do
        sum = sum + input[j] × weights[i][j]
    end for
    output[i] = sum
end for
Return output vector

Algorithm S4: HLS module of Softmax

Input: input vector
Output: output vector

```
# Compute the maximum value
max_val = a[0]
for i = 1 to size − 1 do
    if a[i] > max_val then
        max_val = a[i]
    end if
end for
# Perform exponential operations and compute the sum
sum = 0
for i = 0 to size − 1 do
    a[i] = exp(a[i] − max_val)
    sum = sum + a[i]
end for
# Normalize the values
for i = 0 to size − 1 do
    a[i] = a[i] / sum
end for
Return output vector
```
